# A comment on “Electrodiagnostic criteria for neuromuscular transmission disorders suggested by a European consensus group”

**DOI:** 10.1016/j.cnp.2025.12.009

**Published:** 2026-01-01

**Authors:** Leyla Das Pektezel, Mehmet Yasir Pektezel

**Affiliations:** a Department of Clinical Neurophysiology, Necip Fazil City Hospital, Kahramanmaras, Türkiye; b Department of Neurology, Division of Intensive Care Unit, Necip Fazil City Hospital, Kahramanmaras, Türkiye

**Keywords:** Myasthenia gravis, Seronegative, Electrodiagnosis, Temporary neuromuscular junction dysfunction

Dear Editor,

We read with great interest the article by Tankisi et al. (2025), titled “Electrodiagnostic criteria for neuromuscular transmission disorders suggested by a European consensus group” (Tankisi et al., 2025). Electrophysiological studies play an important role in seronegative myasthenia gravis (MG) patients. As suggested by the ESTEEM Group, at least two electrophysiologic studies should be performed to avoid a misdiagnosis of MG, especially when clinical information is insufficient to reach a final diagnosis. Recently, we examined a 60-year-old male patient who had been referred to the clinical neurophysiology clinic due to generalized weakness. He complained of bilateral ptosis without double vision for the last one month. Generalized weakness in upper and lower extremities had developed for the last two weeks, without any sensory symptoms. There was fluctuation in the symptoms. His medical and family history was unremarkable. Neurological examination revealed significant weakness in both upper and lower extremities (MRC scale: proximal 4/5, distal 5-/5), without any sensory deficit. No evidence of upper motor neuron dysfunction was found, and all deep tendon reflexes were normal.

Nerve conduction studies and electromyography performed on the patient showed completely normal results. Low-frequency repetitive nerve stimulation (RNS) demonstrated a borderline decremental response, with reductions of 9.2 % and 10.4 % in amplitude and area, respectively. The post-exercise decrement was significant, with reductions of 18.3 % in amplitude and 23.4 % in area (Fig.), consistent with findings of post-exercise exhaustion. Post-exercise facilitation was also found. No increment was observed in high-frequency RNS. The AChR antibody level was detected at 0.43 nmol/L, slightly below the positive cut-off (> 0.45 nmol/L). Other laboratory tests were normal such as creatine kinase and thyroid function tests. Chest CT was normal and with no evidence of thymic pathology. Pyridostigmine was started at 180 mg/day. The symptoms demonstrated improvement from the initial dose of treatment and resolved rapidly and almost completely within the first week of the therapy. Three weeks later, single-fiber EMG was planned as the second electrophysiological test, after discontinuing pyridostigmine 12 h prior to the examination. However, the patient did not tolerate the procedure, so RNS was repeated instead. No decremental response was observed in the second RNS following a 12-hour drug-free period. His neurological examination revealed no deficits, simultaneously. RNS was reperformed at 24 and 96 h following a drug-free period. No decremental response or clinical impairment was observed in these examinations. A second electrophysiological test supporting the diagnosis could not be confirmed. The AChR antibody was retested, and its level was 0.01 nmol/L. No previous drug use was reported that could explain this temporary neuromuscular junction dysfunction. Other antibodies—anti-MuSK, anti-LRP4, and anti-titin—were also tested and found to be negative while the patient was followed up off medication. Nerve conduction studies and electromyography were repeated and showed normal findings at the last electrodiagnostic examination. The patient is currently being monitored without any medication and has no symptoms.

**Fig:**
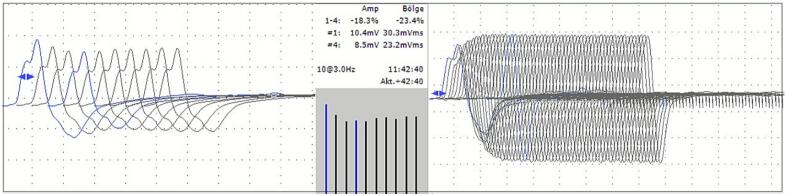
Low-frequency repetitive nerve stimulation (3 Hz-10 stimuli) demonstrates a decremental response in the left abductor digiti minimi during the post-exercise period (Left), and high-frequency repetitive nerve stimulation (30 Hz-60 stimuli) does not show a significant increment (Right). Stimulations are performed at supramaximal intensity and under optimum temperature conditions.

According to the American Association of Neuromuscular & Electrodiagnostic Medicine (AAEM) Guidelines (Medicine AQACAAoE., 2001a, Medicine AQACAAoE., 2001b), the first RNS in this patient, along with the present symptoms and the positive response to pyridostigmine, could support the diagnosis of MG. However, in subsequent RNSs recorded on the abductor digiti minimi and nasalis muscles, no decrement was observed to support the diagnosis of MG based on the ESTEEM Group’s diagnostic criteria, and no clinical impairment was noted during the drug-free period. This clinical follow-up highlights the importance of performing electrophysiological studies more than once, particularly in the patient group defined as seronegative MG, as in ESTEEM Group’s recommendations. The etiology of this temporary neuromuscular junction dysfunction in the patient could not be clarified in a conclusive manner. Although the AChR antibody level did not reach a diagnostic threshold in this patient, a fluctuation near the positive cutoff was observed, and other antibodies were negative. While the clinical significance of this sub-threshold fluctuation is unclear, a change in anti-AChR antibody serum levels has been reported to be associated with clinical status in MG patients (Marcuse et al., 2022). In transient neonatal myasthenia gravis, pathogenic maternal autoantibodies cross the placenta and disrupt neuromuscular junction signaling; the antibodies are subsequently naturally cleared from the infant’s circulation, with a mean symptom duration of 2–3 weeks (Lindroos et al., 2024). On the other hand, the literature includes anecdotal reports of temporary myasthenia gravis–like symptoms associated with various pharmacological agents, chemical exposures, and clinical conditions (Authier et al., 1995, Horrocks and Leonard, 1966, Uludag et al., 2001). Because the diagnosis of seronegative MG is primarily supported by electrophysiological findings, repeating the electrophysiological assessment is crucial to rule out temporary neuromuscular junction dysfunction, such as that observed in our patient. However, we have some concerns regarding the time frame between the tests. A certain interval has not been proposed by the ESTEEM Group. We recommend that a time frame between electrophysiological studies for diagnosing MG be determined. We performed the second study three weeks later, after observing the course. Considering the half-life of both circulating human IgG antibodies and AChRs in a normal, non-denervated neuromuscular junction, an interval of approximately three weeks between electrodiagnostic assessments appears appropriate, regardless of the specific underlying pathology or technical factors (Fichtner et al., 2020, Martinez-Pena and Akaaboune, 2021). Finally, our patient is being followed without any clinical impairment or need for treatment. More attention should be paid, particularly in the process of defining the seronegative MG patient subgroup, which may be at increased risk of overdiagnosis and subsequent unnecessary treatment.

## Declaration of Competing Interest

The authors have no conflicts of interest to declare.
